# Abnormal growth of a pleomorphic leiomyosarcoma originating from the mesenteric vein associated with poor outcome after curative-intent resection: a case report

**DOI:** 10.1186/s40792-022-01497-4

**Published:** 2022-08-01

**Authors:** Masaya Otabe, Tomoyuki Abe, Yusuke Sumi, Shuji Yonehara, Toshio Noriyuki, Masahiro Nakahara

**Affiliations:** 1 Departments of Surgery, Onomichi General Hospital, 1-10-23, Hirahara, Onomichi, Hiroshima 722-8508 Japan; 2 Departments of Pathology, Onomichi General Hospital, Onomichi, Hiroshima Japan

**Keywords:** Leiomyosarcoma, Pleomorphic pattern, Vessel-derived PLMS

## Abstract

**Background:**

A leiomyosarcoma (LMS) is a rare tumor that mainly originates from the urinary tract and digestive system; however, non-visceral organ-derived patterns are rare. Herein, we report that a vessel-derived pleomorphic LMS (PLMS) originating from the mesenteric vein has a poor prognosis even after curative-intent surgery.

**Case presentation:**

The patient was a 41-year-old woman with no relevant medical history. The patient presented with abdominal pain and an abnormal bulge on the left lower abdomen. Magnetic resonance imaging revealed a large tumor occupying the left abdomen. Enhanced computed tomography revealed a bulky tumor with a maximum size of 13 × 13 cm with impending rupture, and a small amount of ascites was detected around the tumor. As the tumor directly invaded the small intestine and descending colon, left hemicolectomy and partial resection of the small intestine were performed. The patient was discharged on postoperative day 10, without any complications. On histopathological analysis, the tumor was diagnosed as a vessel-derived LMS with a pleomorphic pattern. The patient died due to disseminated intravascular coagulation because generalized peritonitis was induced by the super-early recurrence of the tumor 2 months after the surgery.

**Conclusions:**

Regardless of curative-intent surgery for a vessel-derived PLMS, super-early local recurrence and distant metastasis were observed. A vessel-derived PLMS requires further investigation to determine its characteristics and therapeutic strategies to improve long-term prognosis.

## Background

A leiomyosarcoma (LMS) is a relatively rare tumor, accounting for 5–7% of all soft tissue sarcomas [1]. An LMS generally originates from the urinary tract and digestive system; however, non-visceral organ-derived patterns are scarce. By the time an LMS is diagnosed, it could already be large in size owing to the difficulty in early and precise diagnosis. Among several histological classifications of an LMS, a pleomorphic LMS (PLMS) accounts for approximately 8.6% [2]. Although an LMS is often considered an indolent malignancy with an associated good prognosis, a PLMS has an aggressive malignant potential with a dismal prognosis. Risk factors for recurrence-free survival were previously reported to be R1 resection, age, maximal tumor size, other organ invasions, and histologic type [3].

Vessel-derived LMSs are extremely rare; the majority of reported cases involve the inferior vena cava (IVC) originating from smooth muscle cells located in the middle layer of the venous wall. Owing to the rarity of a PLMS originating from the mesenteric vein, its prognosis and recurrence patterns remain unclear. Herein, we report a literature review and case of a vessel-derived PLMS originating from the mesenteric vein following curative resection.

## Case presentation

**Fig. 1:**
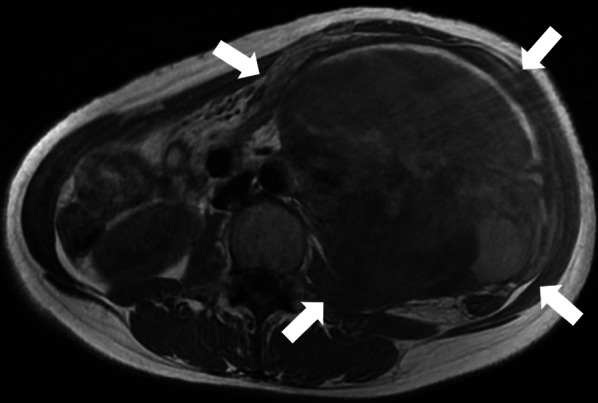
The tumor, with a maximum size of 13 × 13 cm, is located in the left lateral abdomen (arrow). The tumor is uniformly high-intensity with partial low intensity on T2-weighted image

**Fig. 2:**
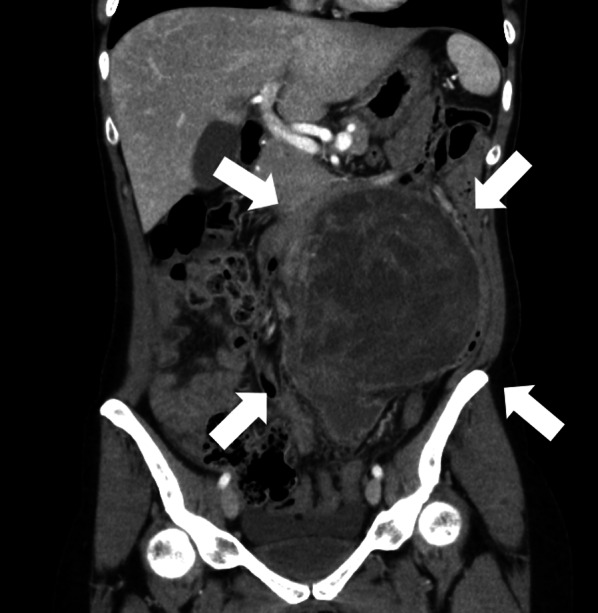
The tumor is located in the upper left abdomen (arrow) and directly invades the descending colon and intestine. Part of the tumor is torn and ascites is detected around the tumor

**Fig. 3:**
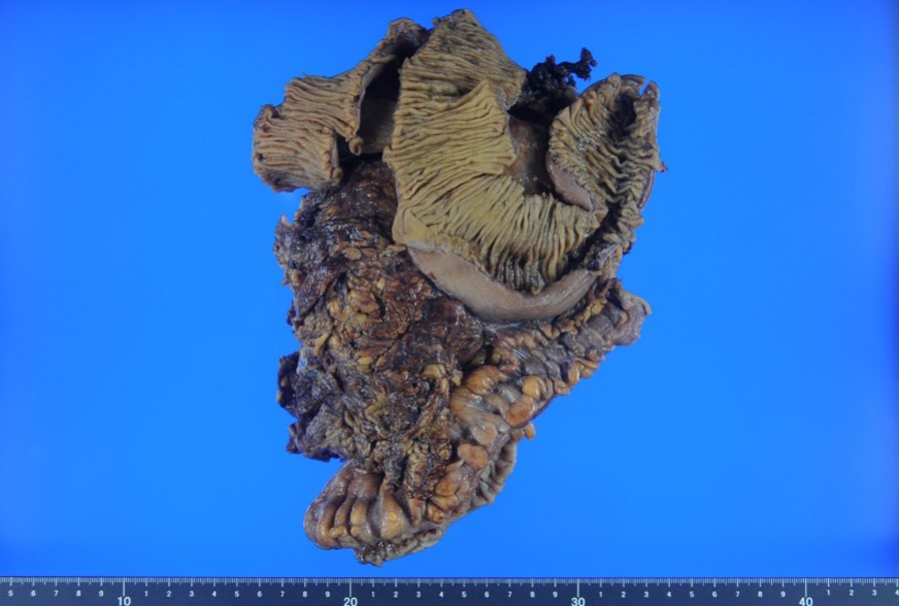
The tumor is predominantly located in the descending colon and colonic mesentery and is hardly included in the intestinal mesentery

**Fig. 4:**
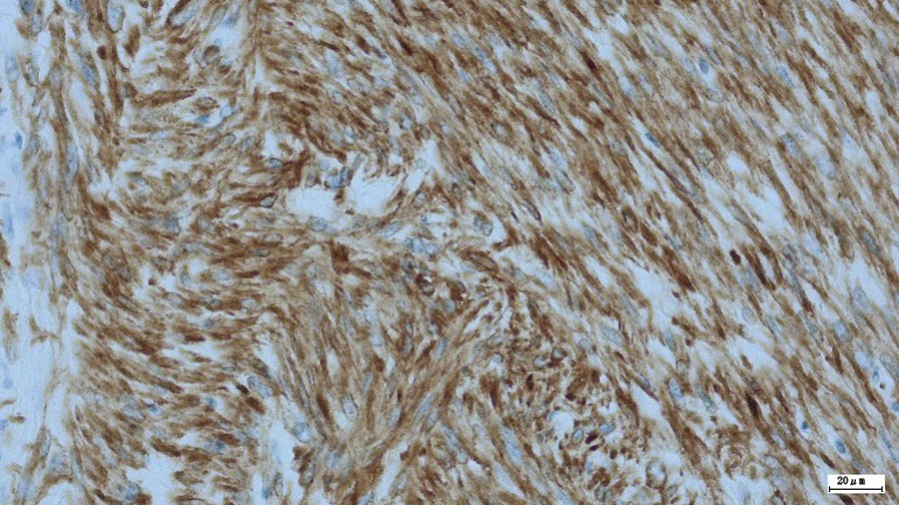
Immunohistochemical analysis reveals that the intravascular tumor is diffusely positive for desmin, but extravascular is negative

**Fig. 5:**
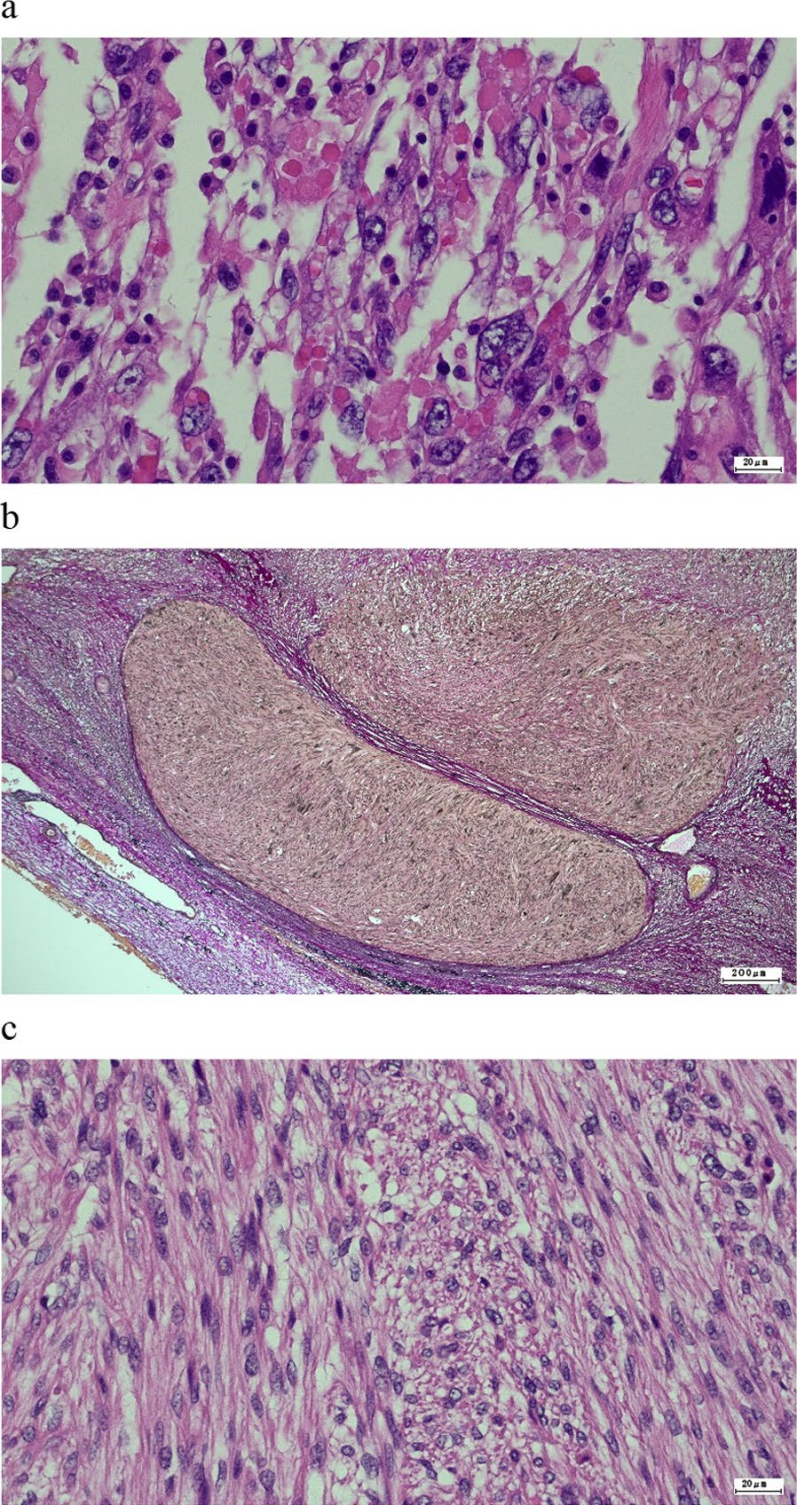
Elastica van Gieson (EVG) staining and hematoxylin–eosin (HE) staining reveal that the majority of the tumor has a pleomorphic pattern (**a**), whereas only intravascular tumor cells of the mesenteric vein are uniformly well-differentiated cells (**b**, **c**)

The patient was a 41-year-old woman with no relevant medical history. The patient’s chief complaint was acute abdominal pain. One week before admission, a bulge in the left lower abdomen was detected and the patient lost her appetite. Laboratory data revealed anemia and elevated C-reactive protein level. Magnetic resonance imaging demonstrated a well circumscribed large tumor, with a maximum size of 13 × 13 cm, occupying the left lateral abdomen (Fig. 1). The tumor directly invaded the small intestine and the descending colon. The tumor showed low intensity on T1-weighted image (WI) and heterogeneous high intensity on T2WI. Contrast-enhanced computed tomography revealed that the intratumor was strongly enhanced in the early phase (Fig. 2). The tumor was suspected to have ruptured because of the irregularity of the tumor surface with a small amount of ascites. The left ureter was dilated because of tumor compression. After admission, a ureteral stent was inserted to prevent intraoperative injury. Severe anemia and localized peritonitis due to rapid progression and laceration of the tumor were observed; therefore, an emergency surgery was performed, on the suspected diagnosis of gastrointestinal stromal tumor or retroperitoneal sarcoma, instead of chemotherapy. Intraoperative findings revealed massive, bloody ascites. The tumor directly invaded the small intestine and descending colon and penetrated the retroperitoneum. A left hemicolectomy and partial resection of the small intestine were performed. The intraoperative bleeding was 1650 mL. The patient was discharged on postoperative day 10, without any postoperative complications. Macroscopically, the tumor was predominantly located in the descending colon and penetrated the retroperitoneum; therefore, the origin of the tumor was believed to be the descending colon (Fig. 3). Microscopically, pleomorphic spindled cells arranged in fascicles intersected at a right angle, and these pleomorphic spindle cells occupied most of the tumor. The nucleus was centrally located, blunt-ended, and appeared to be cigar shaped. Immunohistochemical examination revealed that the cells tested positive for α-smooth muscle actin (α-SMA) and desmin (Fig. 4). On the basis of these findings, the final pathological diagnosis was a PLMS. The results of elastica van Gieson (EVG) and hematoxylin and eosin (HE) staining led to the suspicion that the intravessel well-differentiated tumor originated from the mesocolon (Fig. 5a–c). Therefore, the tumor was considered to have originated from the mesenteric vein.

The patient was scheduled for adjuvant systemic chemotherapy with doxorubicin and ifosfamide; however, she was hospitalized with suspected urinary infection one month after the operation. A large intra-abdominal tumor and ileus due to a recurrent tumor appeared. The patient died 2 months postoperatively because of uncontrolled tumor progression and sepsis.

## Discussion

The abnormal growth of a PLMS prompted us to perform surgery with semi-urgent curative intent. Even after surgery, a large tumor recurrence in the retroperitoneum and lung metastasis were detected. Uncontrollable recurrent tumors can induce sepsis due to colorectal perforations. Notably, on pathological examination, the tumor was a well-differentiated LMS within the mesenteric vein, whereas the majority of tumors had a pleomorphic pattern, particularly in the extravascular region. Based on immunohistochemical analysis, the tumor was highly suspected to be derived from the mesenteric vein. Several studies have reported that the prognostic factors for an LMS are American Joint Committee on Cancer stage, tumor size, and tumor depth [4–8]. However, the rarity of a PLMS makes it difficult to identify prognostic variables after surgery with a curative intent. Considering the poor prognosis of a vessel-derived PLMS, an appropriate treatment strategy for achieving a long-term prognosis is unclear. Currently, curative-intent surgery is considered an essential treatment for a PLMS, regardless of its poor prognosis.

Vessel-derived LMSs account for < 2% of all the LMSs. In general, this type of tumor arises from large vessels such as the IVC, pulmonary vein, and femoral vein [9–11]. Owing to the nature of the vessel-derived LMS, it frequently metastasizes to distant sites, such as the lungs and liver via the vessel stream [10–13]. Previously, the rates of 5-year disease-free survival and overall survival (OS) were reported to be approximately 40% and 75%, respectively, in cases of an LMS derived from IVC [14]. Due to the scarcity of cases of LMSs derived from the mesenteric vein, prognosis is dependent on the tumor differentiation of an LMS. The pleomorphic type, tumor size (≥ 50 mm), and an advanced stage are regarded as risk factors for long-term prognosis. Considering that our patient had several risk factors for survival, rapid progression might have occurred even after resection with curative intent. To the best of our knowledge, this is the first case of a vessel-derived LMS arising from the mesenteric vein. It may delay diagnosis because of its deep and intravascular location [12, 15]. The tumor tends to grow and occlude vessels and collateral circulation even after complete occlusion. Most cases are diagnosed at advanced stages owing to a lack of surveillance and asymptomatic state [12, 16, 17]. In this case, the origin was suspected to be the mesenteric vein and the tumor size was already large when it was reported. Lung metastasis occurred 1 month after surgery, and a large intra-abdominal tumor appeared.

On pathological examination, the intravascular tumor was well differentiated, whereas most tumors had a pleomorphic pattern. According to the tumor growth pattern, it was presumed that the tumor infiltrated and proliferated outside the blood vessels as the tumor grew. The tumor originating from the intravascular LMS showed giant cells with strong nuclear atypia and spindle-shaped cells, consistent with the pleomorphic pattern. EVG and HE staining revealed that the intravessel tumor was well-differentiated.

The typical LMS component was positive for myogenic markers α-SMA, desmin, H-caldesmon, and calponin. The PLMS component was positive for myogenic markers. However, the positive ratio of myogenic markers for a PLMS was lower than that for an LMS. The MIB-1 labeling index in a PLMS was significantly higher than that in an ordinary LMS. In our case, α-SMA and desmin were positive, and pleomorphic spindled cells arranged in fascicles intersected at a right angle. The nucleus was centrally located and blunt-ended, and it appeared to be cigar shaped.

Neoadjuvant systemic chemotherapy, such as doxorubicin alone or in combination with ifosfamide or radiotherapy (RT), did not improve OS in patients with LMSs. To date, perioperative systemic chemotherapy and RT have not conquered the survival benefits of complete resection. Further studies are required to prolong the OS of patients with an LMS. Treatment methods for LMSs other than surgery have not yet shown any clear benefits; therefore, complete resection is the only established therapy. However, there are some trials for this [18, 19]. Determining the treatment for LMSs is complex and cases should be discussed among multidisciplinary team members.

## Conclusion

In conclusion, we report a case of a vessel-derived PLMS with a poor prognosis due to aggressive tumor growth. A vessel-derived PLMS requires further investigation to determine its characteristics and therapeutic strategies to improve long-term prognosis.

## Data Availability

Data sharing is not applicable to this article, as no datasets were generated or analyzed during the current study.

## Abbreviations

LMS: Leiomyosarcoma
PLMS: Pleomorphic leiomyosarcoma
IVC: Inferior vena cava
α-SMA: α-Smooth mesenteric actin
EVG: Elastica van Gieson
HE: Hematoxylin–eosin
RT: Radiotherapy
OS: Overall survival
